# CpLEPA Is Critical for Chloroplast Protein Synthesis Under Suboptimal Conditions in *Arabidopsis thaliana*

**DOI:** 10.1371/journal.pone.0049746

**Published:** 2012-11-15

**Authors:** Dai-Li Ji, Hong Lin, Wei Chi, Li-Xin Zhang

**Affiliations:** Photosynthesis Research Center, Key Laboratory of Photobiology, Institute of Botany, Chinese Academy of Sciences, Beijing, China; Iowa State University, United States of America

## Abstract

LEPA is one of the most conserved translation factors and is found from bacteria to higher plants. However, the physiological function of the chloroplast LEPA homolog in higher plants remains unknown. Herein, we demonstrate the physiological role of cpLEPA in enabling efficient photosynthesis in higher plants. The *cplepa-1* mutant displays slightly high chlorophyll fluorescence and pale green phenotypes under normal growth conditions. The growth of the *cplepa-1* mutant is reduced when grown on soil, and greater reduction is observed under intense light illumination. Photosynthetic activity is impaired in the *cplepa-1* mutants, which is reflected in the decreased steady-state levels of chloroplast proteins. *In vivo* protein labeling experiments explained the decrease in the steady-state levels of chloroplast proteins. An abnormal association of the chloroplast-encoded mRNAs with ribosomes suggests that the protein synthesis deficiencies in *cplepa-1* are due to defects in translation initiation in the chloroplasts. The cpLEPA protein appears to be an essential translation factor that promotes the efficiency of chloroplast protein synthesis.

## Introduction

LEPA is one of the most conserved proteins, and it has the unexpected ability to back-translocate tRNAs on the ribosome [1]. LEPA homologs are highly conserved in terms of both their structure and their amino acid sequence, and they are found in bacteria, mitochondria and chloroplasts, but not in archaea or in the cytoplasm of eukaryotes [1]. Based on the domain definition of EF-G, LEPA can be divided into five domains, four out of the five EF-G domains–I, II, III, and V–are present in LEPA. Domain IV and the G′ subdomain of domain I of EF-G are absent. LEPA has a special C-terminal domain called CTD with an unusual fold which might interact with tRNA or 23S rRNA [2].

Although the overall structure of LEPA has been described in great detail, the physiological functions involved in translation have not yet been resolved. In *E. coli*, *LEPA* is located upstream of the *LEP* gene, which encodes nonspecific signal peptidase I [3]. Deletion of LEPA does not cause any apparent phenotype under optimal growth conditions [4], [5]. These observations are difficult to reconcile with the ubiquity of LEPA and its extreme conservation. Other results have demonstrated that, although *E. coli* LEPA-defective cells grown in rich medium have no phenotype [4], under several stress conditions, including high salt, low pH, and low temperature, the *LEPA* mutant is overgrown by wild-type bacterial cells [6]. In bacteria, ΔLEPA strains have been shown to be hypersensitive to potassium tellurite and penicillin [7] and to enhance the production of the calcium-dependent antibiotic in *Streptomyces* bacteria [8]. Recent studies suggested that LEPA may react with both the PRE and POST ribosome complexes, leading to the formation of an intermediate complex that effectively sequesters a catalytically active ribosome, resulting in a transient inhibition of elongation that provides a mechanism for the optimization of functional protein synthesis [9], [10].

The physiological function of the chloroplast homologs of LEPA (cpLEPA) *in vivo* has not been characterized. In this study, we report the identification of an *Arabidopsis* ΔLEPA mutant, which was termed *cplepa-1.* A slightly high chlorophyll fluorescence and pale green phenotype are detected in the *cplepa-1*mutant when grown under normal growth conditions. Physiological and biochemical analyses of the mutant revealed that cpLEPA has an important function in chloroplast biogenesis and plays an essential role in chloroplast translation.

## Results

### Chloroplast LEPA in *Arabidopsis* is a Highly Conserved Homolog of EF-G

**Figure 1:**
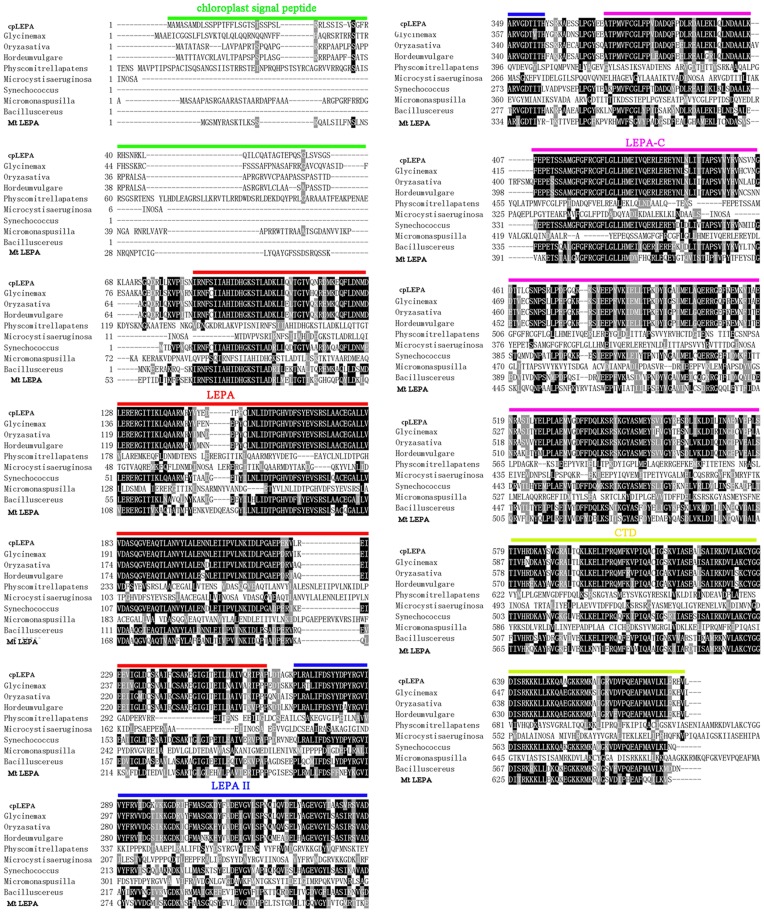
CpLEPA Protein Sequence Alignment. The amino acid sequence of cpLEPA was compared with the sequences of homologous proteins from mitochondria in *Arabidopsis*, *Oryza sativa, Glycine max, Physcomitrella patens*, *Hordeum vulgare, Micromonas pusilla, Synechococcus, Microcystis aeruginosa,* and *Bacillus cereus*. The black boxes indicate strictly conserved amino acids, and the gray boxes indicate closely related residues. The predicted chloroplast transmembrane peptides are underlined in green, The LEPA domains are underlined in red, and the LEPA-II domain is underlined in blue. LEPA-C is underlined in purple, and the CTD is underlined in yellow.

Database searches and protein sequence alignments revealed that cpLEPA shares significant sequence identity with its homologs, from bacteria to eukaryotes (64%–87%) (Figure 1). *CpLEPA* encodes a 681-amino acid protein with a calculated molecular mass of 75 kD. This protein was predicted to be localized to the chloroplast, and the N-terminal 51 amino acids were predicted to be a chloroplast transit peptide by the programs TargetP 1.1 and ChloroP 1.1 (Figure 1). Analysis by the TMHMM program suggests that cpLEPA does not contain a transmembrane domain (data not shown). Four out of the five CpLEPA domains share strong similarity to the counterpart of EF–G, except for domain IV, whereas the CTD is unique to cpLEPA (Figure 1).

### CpLEPA is Associated with the Thylakoid Membrane

**Figure 2:**
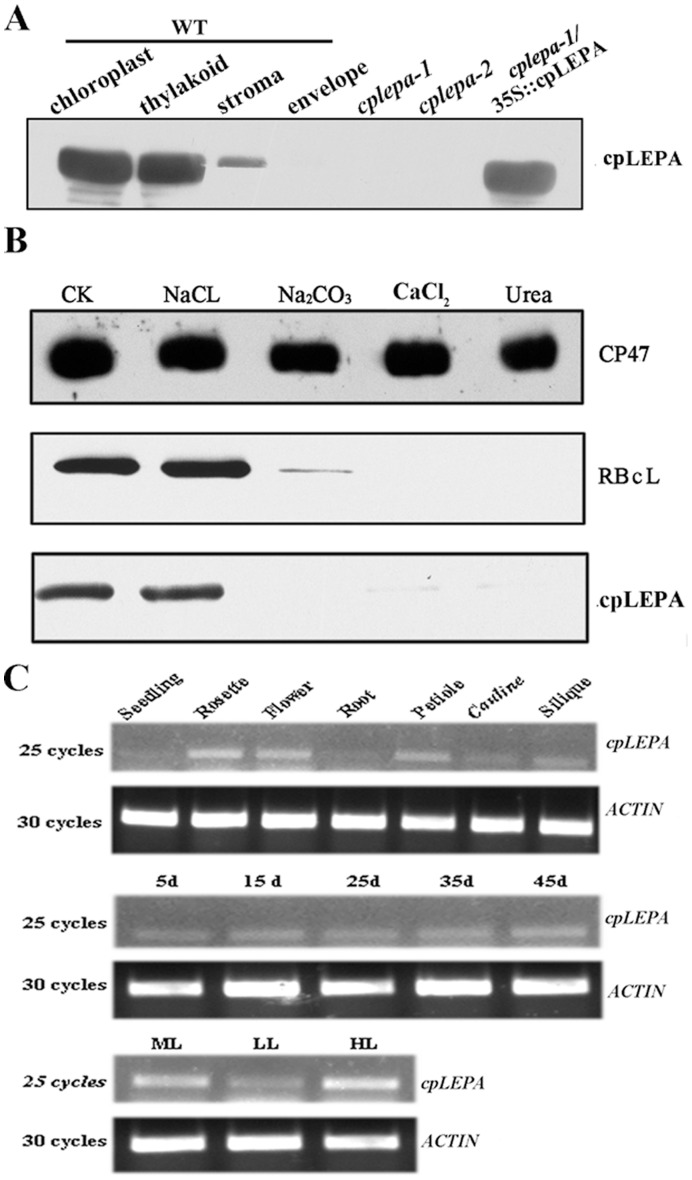
Immunolocalization and Expression of *cpLEPA*. A: Immunolocalization analysis of cpLEPA. The chloroplast, thylakoid, stroma and envelope fractions were subjected to immunoblot analysis with specific antisera against cpLEPA. Equal amounts of protein (20 µg) were loaded in each lane. The lanes marked *cplepa-1*, *cplepa-2* and *cplepa-1/*35s::cpLEPA were loaded with equal amounts of total protein (20 µg). B: Salt washing of the membranes. The thylakoid membranes were incubated with 250 mM NaCl, 200 mM Na_2_CO_3_, 1 M CaCl_2_ and 6 M urea for 30 min at 4°C. Then, the thylakoid proteins were separated by SDS-PAGE and immunoblotted with anti-LEPA, anti-RbcL (ribulose-1,5-bisphosphate carboxylase/oxygenase large subunit) and anti-CP47 antibodies. RbcL and CP47 were used as markers. Thylakoid membrane preparations that had not been subjected to treatment were used as controls. C: Expression patterns of *cpLEPA.* Upper panel: *cpLEPA* expression levels in different organs of *Arabidopsis*, as determined by RT-PCR analysis. RNA samples isolated from seedlings, rosettes, flowers, roots, petiole, cauline tissue and siliques of wild-type plants were reverse-transcribed and subjected to PCR analysis. Middle panel: Transcript levels of *cpLEPA* in *Arabidopsis* leaves at 5, 15, 25, 35 and 45 d. Bottom panel: Light-induced accumulation of *cpLEPA* transcripts. Three-week-old plants grown under medium light (120 µmol m^−2^ s^−1^), low light (40 µmol m^−2^ s^−1^) or high light (500 µmol m^−2^ s^−1^) were used. *ACTIN* is shown as a control.

To investigate the localization of cpLEPA, intact chloroplasts were isolated and fractionated, and the proteins were subjected to immunoblot analysis with a specific cpLEPA antibody. Under normal growth conditions (120 µmol m^−2^ s^−1^), most of the cpLEPA protein was detected in the thylakoid fractions (Figure 2A), and the ratio of cpLEPA in the stroma to cpLEPA in the thylakoid membrane was approximately 0.25. These results indicate that cpLEPA is a membrane-associated protein. To further investigate the degree of membrane association of cpLEPA, we treated the thylakoid membrane with salts and chaotropic agents. Washing the membrane with 0.25 M NaCl did not release the cpLEPA from the membrane, but cpLEPA was barely detectable after washing the membrane with 0.2 M Na_2_CO_3_, 1 M CaCl_2_, or 6 M urea. As a control, the integral membrane protein CP47 was not released from the membranes by such treatments. RBcL, which is located in the stroma and thylakoid membrane, yielded results similar to those of cpLEPA (Figure 2B).

*CpLEPA* is widely expressed in most *Arabidopsis* green tissues, including the seedlings, leaves, stems, siliques, flowers and cauline tissue (not in the roots), but the expression levels of c*pLEPA* in seedlings and cauline tissue are reduced compared with those in leaves, as revealed by RT-PCR analysis (Figure 2C). The expression of *cpLEPA* appeared to be independent of the age and developmental stage of the *Arabidopsis* leaves (Figure 2C). To examine the effects of light intensity on the expression of *cpLEPA* in *Arabidopsis*, the level of *cpLEPA* transcripts in leaves grown under normal light was compared with the level in plants grown under high light and low light. Relative to normal growth conditions, the *cpLEPA* transcript level was decreased under low light and increased under high light conditions (Figure 2C).

### Knock-out of CpLEPA Leads to Growth Retardation and Impaired Chloroplast Development

**Figure 3:**
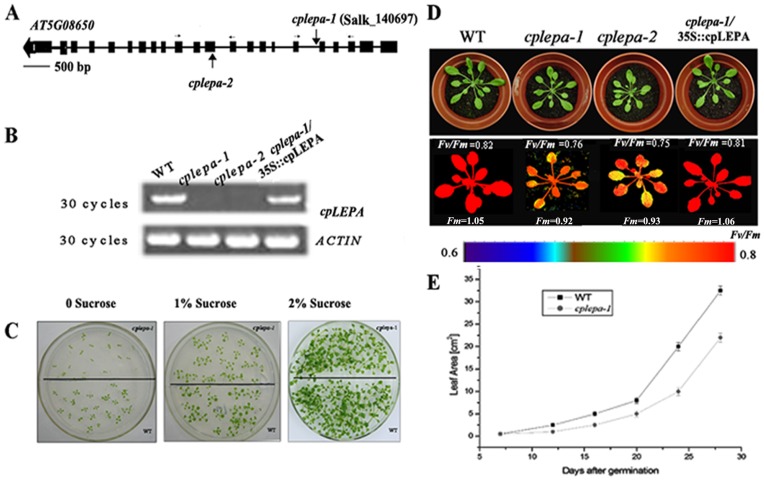
Identification and Phenotyping of the *cplepa* Mutants. A: T-DNA insertion sites in the *cpLEPA* gene. Exons are indicated by black boxes, introns by lines, and the T-DNA insertions by vertical arrows. The horizontal arrows illustrate the primers used for T-DNA insertion verification and RT-PCR. The scale bar indicates 500 bp. B: RT-PCR analysis. RT-PCR was performed using specific primers for *cpLEPA* or *ACTIN*. C: Two-week-old WT and *cplepa-1* mutants grown on MS medium supplied with 0, 1% sucrose and 2% sucrose. D: Complementation of the *cplepa-1* mutant. The cDNA of the *cpLEPA* gene was cloned into a binary plant transformation vector and used for complementation of the *cplepa-1* mutant (*cplepa-1*/35S::*cpLEPA*). Four-week-old WT, *cplepa-1, cplepa-2* and *cplepa-1*/35S::*cpLEPA* plants were grown on soil. Fluorescence was measured with a CF Imager and visualized using a pseudocolor index, as indicated at the bottom, *Fm* and *Fv/Fm* value were presented. E: Growth of wild-type and *cplepa-1* mutant plants on soil at 120 µmol m^−2^ s^−1^. The values shown are averages ± s.e. (n = 6).

To examine the function of cpLEPA in plants, we obtained two *cplepa* mutants from ABRC. The mutants contain T-DNA insertions within the fifth intron and the eleventh exon of the *cpLEPA* gene and are termed *cplepa-1* and *cplepa-2*, respectively (Figure 3A). The T-DNA insertions were confirmed by PCR and subsequent sequencing of the amplified products. RT-PCR analysis revealed that expression of the *cpLEPA* gene was undetectable in the *cplepa-1* and *cplepa-2* mutants (Figure 3B). Immunoblot analysis showed that the cpLEPA protein was undetectable in the *cplepa* mutants, and the protein levels of cpLEPA in the complemented mutant plants were comparable to those of wild-type plants (Figure 2A).

The *cplepa-1* and *cplepa-2* mutants showed no growth differences compared with wild-type plants when grown on solid MS medium supplied with 2% sucrose at 120 µmol m^−2^ s^−1^ light illumination. When the sucrose was decreased to 1% or without sucrose, the growth of *cplepa-1* was greatly retarded (Figure 3C). When grown on soil, the *cplepa-1* and *cplepa-2* mutant plants displayed slightly high chlorophyll fluorescence and a pale green phenotype (Figure 3D). Chlorophyll fluorescence analysis of dark-adapted leaves of wild-type and mutant plants showed that the *F_v_/F_m_* ratio was slightly lower (0.76±0.01) in the *cplepa-1* plants than in the wild-type plants (0.81±0.01). As shown in Figure 3E, the leaf area of the mutant was approximately 70% that of the wild-type plants 28 d after germination. The chlorophyll content in *cplepa-1* was reduced to approximately 88% of the wild-type level, and chlorophyll *a*/*b* was decreased to approximately 2.6, compared with 3.0 in the wild-type (Table 1).

**Table 1:** Chlorophyll Contents in Wild-Type and *cplepa-1* Plants.

chlorophyll	WT	*cplepa-1*	*cplepa-1*/WT(%)
Chl *a*	792.6±17	699.6±12	88
Chl *b*	261.4±12	261.1±11	100
Chl *a*+*b*	1083.8±31	963.7±14	89
Chl *a*/Chl *b*	3±0.1	2.6±0.1	87

To examine the effects of cpLEPA deletion on the chloroplast ultrastructure, we analyzed electron micrographs of ultrathin sections from 3-week-old leaves of wild-type and mutant plants. In total, 100 chloroplasts were examined, and the micrographs showed that the wild-type chloroplasts have well-structured thylakoid membrane systems, whereas the *cplepa-1* chloroplasts are smaller (7.0±0.6 and 6.3±0.3 µm, respectively). In addition, *cplepa-1* has fewer discs per grana stack (12±1 and 7±2, respectively) and exhibits disrupted thylakoid membrane organization, which further suggests that the cpLEPA mutation affects chloroplast development (Figure S1).

### Accumulation and Synthesis of Chloroplast Proteins in *cplepa-1*

**Figure 4:**
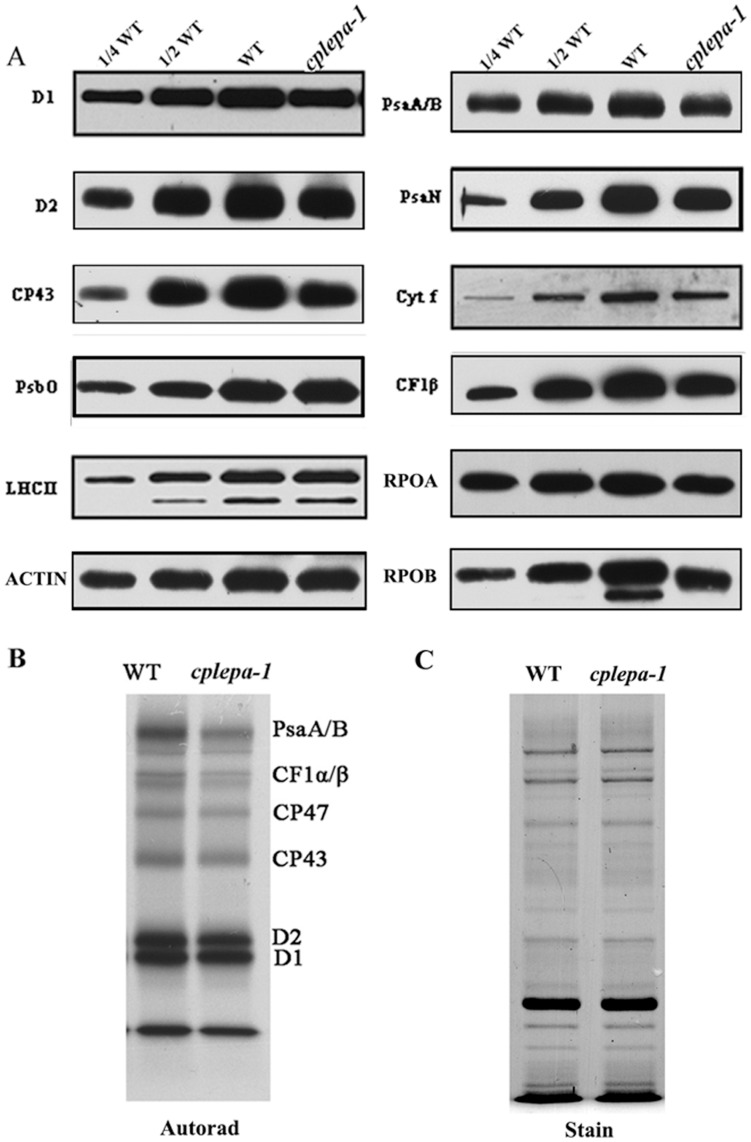
Accumulation and Synthesis of Chloroplast Proteins in *cplepa-1* Plants. A:Immunoblot analysis of total protein extracts from wild-type and *cplepa-1* plants. Wild-type and *cplepa-1* plants grown on soil at a photon flux density of 120 µmol m^−2^ s^−1^ were used. For wild-type and *cplepa-1* plants, 10 µg of total proteins were loaded. The antibodies used are indicated on the right. Actin served as a control to normalize the protein levels. Similar results were obtained in two additional independent experiments. B: Pulse labeling of thylakoid proteins. Primary leaves of 12-day-old plants were radiolabeled with [^35^] S-methionine in the presence of cycloheximide for 20 min. The thylakoid membranes were isolated, separated by SDS-urea-PAGE and visualized autoradiographically, lanes were loaded with equal protein contend. C: A coomassie blue-stained gel is presented to show that equal amounts of proteins were loaded.

The levels of chloroplast proteins were examined in the *cplepa-1* mutant using specific antibodies. The levels of PEP-dependent plastid-encoded chloroplast proteins were reduced. Subunits of PSI (PsaA/B, PsaN) and PSII (D1, D2 and CP43), Cytf of the cytochrome *b_6_f* complex and the β-subunit of the chloroplast ATP synthase accumulated to approximately 60–70% of their wild-type levels in *cplepa-1*. In contrast, the accumulation of nuclear-encoded PsbO and LHCII was not affected in the *cplepa-1* mutant (Figure 4A).

To investigate the possibility of diminished accumulation of chloroplast proteins, we first studied the synthesis of thylakoid proteins by *in vivo* pulse-chase labeling experiments. For these experiments, the leaf proteins were pulse labeled with [^35^S]-Met in the presence of cycloheximide, which blocks the synthesis of nuclear-encoded proteins. As shown in Figure 4B, the rates of synthesis of the PSI reaction center PsaA/B; the PSII reaction center D1, D2, CP47 and CP43; and the α- and β-subunits of the chloroplast ATP synthase (CF1-α/β) were reduced to 60–70% of wild-type levels (Figure 4B).

### Chloroplast mRNAs are Associated with Abnormally Few Ribosomes in *cplepa-1*

**Figure 5:**
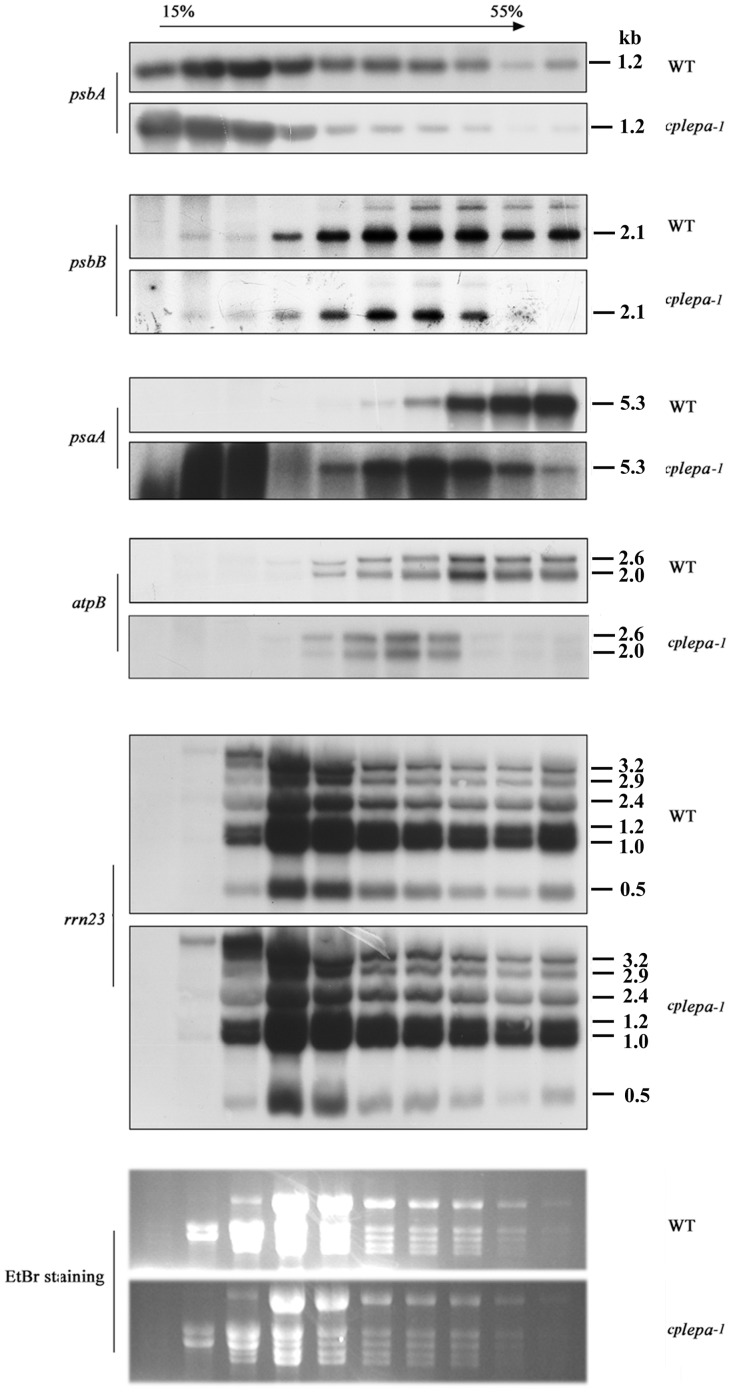
Polysome Association Analysis for Chloroplast Transcripts in Wild-Type and *cplepa-1* Plants. The association of *psbA*, *psbB*, *atpB*, *psaA* and *rrn23* transcripts with polysomes. Total extracts from wild-type and *cplepa-1* leaves grown on soil for 3 weeks at 120 µmol m^−2^ s^−1^ were fractionated on 15%–55% sucrose gradients. Ten fractions of equal volume were collected from the top to the bottom of the sucrose gradients, and equal proportions of the RNA purified from each fraction were analyzed by northern-blot analysis. The rRNAs were detected by ethidium bromide (EtBr) staining. The size of the transcript (in kb) is shown.

The chloroplast protein synthesis deficiency in the *cplepa-1* mutant prompted us to examine the association of chloroplast mRNA with ribosomes. Polysome analysis provides an estimate of the efficiency of translation initiation and elongation [10]. Polysome association was conducted using three-week-old plants grown on soil and MS solid medium. Total leaf lysate was fractionated on a sucrose gradient under conditions that preserve polysome integrity, and mRNAs purified from the gradient fractions were identified by hybridization with specific probes. The PEP-dependent plastid-encoded *atpB, psbA, psbB,* and *psaA/B* transcripts showed a small shift toward the top of the gradient in the *cplepa-1* mutant grown on soil (Figure 5). However, the distribution of mutant and wild-type plastid 23S rRNA, *ndhA*, *petA* and *psaJ* transcripts were unchanged in the sucrose gradients (Figures 5 and S2). In addition, the distribution of 23S rRNA along the sucrose gradients showed a different sensitivity to EDTA compared to that of *rbcL* mRNA (Figure S2). When grown on MS, however, no significant differences in sedimentation between wild-type and mutant plants were detected for the *atpB, psbA, psbB, petB,* or *psaA/B* mRNAs (Figure S3). These results indicated that, in the soil-grown *cplepa-1* mutant, chloroplast protein translation was impaired.

### PEP-dependent Chloroplast-encoded mRNA Transcripts are Dramatically Reduced in *cplepa-1*

**Figure 6:**
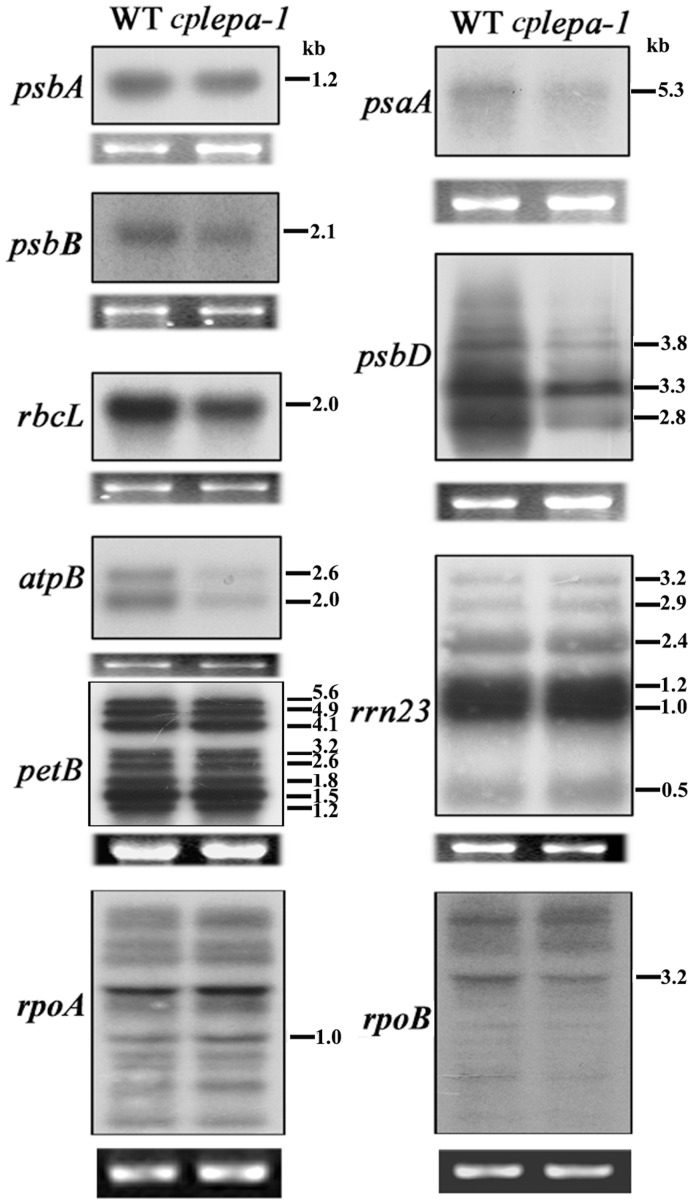
Northern Blot Analysis for Chloroplast Transcripts in Wild-Type and *cplepa-1* Plants. Northern blot analysis of the chloroplast transcripts *psbA*, *psbB*, *psbD*, *atpB*, *psaA*, *petB*, *rbcL*, *rpoA*, *rpoB* and *rrn23* in wild-type and *cplepa-1* mutant plants. Each lane was loaded with 10 µg of total RNA. The plants were grown on soil for 3 weeks under 120 µmol m^−2^ s^−1^ illumination. Additionally, 25S rRNA stained with EtBr was loaded as a control. The size of the transcript (in kb) is shown.

The levels of the chloroplast-encoded transcripts were also investigated by RNA gel blot hybridization using the same material as described in the polysome association experiments. Our results showed that the levels of mRNAs encoding the PsaA subunit of PSI (*psaA-psaB-rps14)* were reduced to 20% of wild-type levels in the mutant (Figure 6). Except for 23s rRNA, an approximately two fold decrease was also observed in the levels of transcripts encoding the following photosynthetic proteins: D1 (*psbA*), CP47 (*psbB-psbT-psbH-petB-petD*), D2 (*psbD-psbC*), atpB (*CF1* β), and RBcL (*rbcL*) (Figure 6). The levels of chloroplast transcripts examined were not affected in the mutant plants when grown on MS (Figure S4).

### Increased Sensitivity of the *cplepa* Mutants to High Light

**Figure 7:**
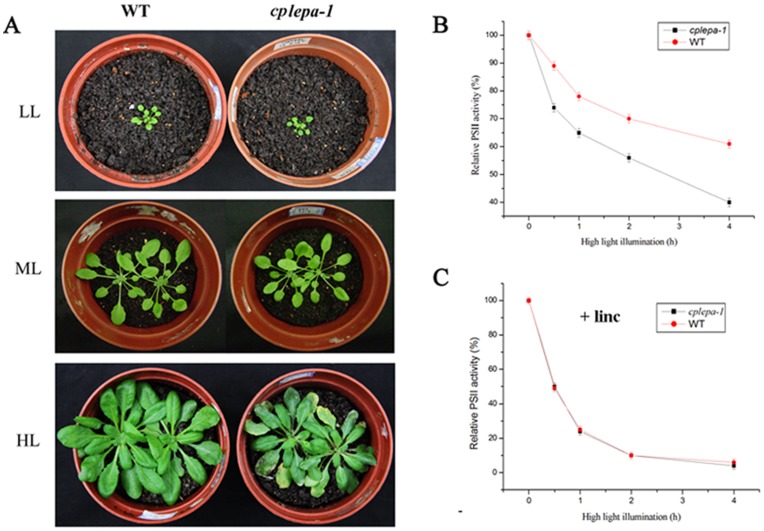
Photosensitivity Analysis of *cplepa-1* Plants. A: The phenotypes of wild-type (WT) and *cplepa-1* mutant plants grown in a growth chamber at 120 µmol m^−2^ s^−1^ in the first two weeks, then transferred to low light (40 µmol m^−2^ s^−1^) or high light (500 µmol m^−2^ s^−1^) for another two weeks. B: The *Fv/Fm* ratio was measured for detached leaves from wild-type (WT) plants (red circles) and *cplepa-1* mutant plants (black squares) following high-light illumination (1,000 µmol m^−2^ s^−1^) in the absence of lincomycin (Lin). C: The *Fv/Fm* ratio was measured for detached leaves from wild-type (WT) plants (red circles) and *cplepa-1* mutant plants (black squares) following high-light illumination (1,000 µmol m^−2^ s^−1^) in the presence of lincomycin (Lin).

When wild-type and *cplepa-1* mutant plants that were initially grown at 120 µmol m^−2^ s^−1^ were transferred to low-light and high-light growth conditions for another two weeks, the growth of the mutants was greatly inhibited under high light. The mutants did not differ from the wild-type plants under low light (Figure 7A). To further determine whether the *cplepa-1* mutant is sensitive to high light, *Fv/Fm* was measured in the wild-type and *cplepa-1* plants under high-light illumination of 1,000 µmol m^−2^ s^−1^. In the absence of lincomycin, within 2 h of illumination at a light intensity of 1,000 µmol m^−2^ s^−1^, *Fv/Fm* declined in the wild-type and mutant leaves to approximately 73% and 55% of the dark-adapted values, respectively. After 4 h of illumination, *Fv/Fm* declined in the wild-type and mutant leaves to approximately 60% and 40% of the dark-adapted values, respectively (Figure 7B). These results clearly demonstrated the increased photosensitivity of the mutants. In the presence of lincomycin, the decrease in *Fv/Fm* was more rapid and continued until the *Fv/Fm* values approached approximately 10% of the dark-adapted values in wild-type leaves (Figure 7C). In the presence of lincomycin, the decline in *Fv/Fm* in the *cplepa* mutants was similar to that observed in the wild-type leaves during the same photoinhibitory light treatment (Figure 7C). Because lincomycin blocks the repair of PSII by inhibiting *de novo* chloroplast protein synthesis, these results suggest that the mutant’s sensitivity to high light is due to an impaired PSII repair process.

## Discussion

LEPA is an extremely conserved and widely distributed translation factor [11]. The amino acid sequence of *Arabidopsis* cpLEPA shows 64% amino acid identity with that of *E. coli* LEPA (Figure 1). This degree of sequence conservation is particularly high for a comparison between higher plants and bacteria. CpLEPA contains four domains: LEPA, LEPA-II, LEPA-C and a CTD domain. The LEPA and LEPA-II domains contain the extremely conserved key amino acids that are important for GTP binding, which are known as the G1, G2, G3 and G4 sequence motifs. The G1, G3 and G4 motifs are responsible for binding and hydrolyzing GTP and for interacting with the cofactor Mg^2+^ [12]. The G2 motif undergoes a conformational change that is essential for GTPase function [13]. LEPA-C was predicted to function in translation elongation. The structure and sequence similarity of cpLEPA to *E. coli* LEPA indicates a role for this protein in the efficiency of chloroplast protein translation.

LEPA was initially reported as the leader peptide of the *lep* operon and was described as a membrane-associated GTP-binding protein [4]. The N-terminal 51 amino acids of *Arabidopsis* cpLEPA was hypothesized to function as a chloroplast signal peptide (Figure 1). Immunoblot analysis verified that cpLEPA is located in the chloroplast and is primarily found in association with the thylakoid membrane. Membrane-associated cpLEPA could be washed out by Na_2_CO_3_ and CaCl_2,_ indicating that cpLEPA is not an integral membrane protein and that the association with the membrane is flexible (Figure 2). Pech *et al* suggested that the membrane acts as a storage depot for LEPA and that LEPA is released into the cytoplasm as needed under specific stress conditions in *E. coli* [5]. Considering the association of cpLEPA with the thylakoid membrane, such an arrangement might facilitate the production of functional protein under different stress conditions.

We also observed no growth differences between the *cplepa-1* mutants and wild-type plants when grown on MS medium supplied with 2% sucrose under a light intensity of 120 µmol m^−2^ s^−1^ (Figure 3C). However, the growth of *cplepa-1* was greatly retarded on MS medium supplied with 1% sucrose or without sucrose under the same light intensity (Figure 3C). Sucrose is an important nutrient which affects overall plant growth features. Plant makes and transports sucrose for store or for use through photosynthesis activity. If photosynthesis was impaired, sucrose starvation will greatly decrease plant growth [14]. In addition, the growth of the *cplepa-1* mutant is reduced when grown on soil, and the reduction is increased under high light illumination (Figure 7A). Moreover, the *cplepa-1* mutant shows a slightly pale green phenotype and impaired chloroplast development (Figure S1). PSII and PSI activities are also decreased when grown on soil. These results indicate that, although cpLEPA is not essential under optimal conditions, it becomes critical under nutrient limitation or light stress conditions. PSII activity, indicated by the *Fv/Fm* value, revealed enhanced sensitivity to high-light treatment in the *cplepa-1* mutant in the absence of lincomycin compared with the wild-type plants. The rate of PSII photoinhibition was similar in the mutant and wild-type plants in the presence of the protein synthesis inhibitor lincomycin (Figure 7B, C). The adverse effect of high light on the *cplepa-1* mutant indicates that the repair of PSII was perturbed. Thus, cpLEPA might be involved in the regulation of the synthesis of PSII proteins.

The association of the chloroplast-encoded *psbA*, *psbB*, *psaA/psaB* and *atpB* mRNAs with ribosomes in the mutant grown on soil showed a small shift toward the top of the gradient in the ribosome loading assay (Figure 5), this indicated that translation initiation was impaired in these transcripts. However, the distribution of mutant and wild type plastid 23S rRNA, *ndhA*, *petA* and *psaJ* transcripts were unchanged in the sucrose gradients (Figure S2B). Further exploration of the distribution of polysome association revealed that 23S rRNA displayed a different sensitivity to EDTA compared with *rbcL* mRNA (Figure S2A). It is likely that a significant proportion of the 23S rRNA is found in ribonucleoprotein complexes other than polysomes. Alternatively the ribosomes on which these chloroplast mRNAs are translated represent only a small part of the total ribosome pool (Figure 5). The steady-state transcript levels of PEP-dependent genes, including *psbA*, *psbB, rbcL*, *psaA*, *atpB* and *psbD*, decreased drastically in *cplepa-1* mutants grown on soil (Figure 6). Changes in chloroplast translation might modulate the stability of a subset of chloroplast mRNA molecules [11], [15]. The inactivation of AtprfB affects the polysomal association of the *atpE* transcript and leads to a 50% reduction in the amount of *atpE* transcripts [16]. In *apg3-1*, the abnormal polysomal association of UAG-containing transcripts leads to decreased stability of the transcripts [17]. In *hcf173*, the decreased ribosomal loading of the *psbA* transcript affects the stability of the *psbA* transcript and leads to a significant reduction in its steady-state level [18]. In addition, decreased protein levels of RPOA and RPOB (the α- and β- subunits of PEP) were observed in the *cplepa* mutant (Figure 4A). Thus, it is likely that the dramatic loss in chloroplast transcripts observed in the *cplepa* mutant might be the synergistic effect of decreased chloroplast translation and decreased PEP transcription.

Photosynthetic activity is somewhat impaired in *cplepa-1* mutants, which is reflected in the decreased steady-state level of chloroplast proteins (Figure 4A). Although a dramatic loss in chloroplast transcripts and a perturbation in chloroplast polysome loading were observed in the *cplepA* mutant, only an approximate 20% decrease was observed in the steady-state levels of the proteins. One possibility is that chloroplast genes are transcribed in excess [19]. The *rpoA* mRNA levels are 30-fold higher than the *rpoB* mRNA levels, but the steady-state protein level of RpoB is approximately 50% of that of RpoA [20], [21]. Similarly, the *psbA* mRNA levels are fivefold greater than those of the *psaA-psaB* transcripts because of the increased turnover rate of *psbA* needed to maintain normal photosynthetic activity, whereas the protein levels of these genes remain similar [22], [23]. Polysomes analysis provides an estimate of the efficiency of translation initiation and elongation [11]. There was a relative increase in nonpolysomal chloroplast mRNAs in the *cps2* mutant, but a substantial fraction of mRNAs still remained associated with multiple ribosomes [11]. In this mutant, chloroplast protein translation was only very mildly affected. The effects of the *cpLEPA* mutation on the association of the *psbA*, *psbB*, *atpB*, and *psaA/B* mRNAs with ribosomes were similar to those of *cps2* [11] (Figure 5). *In vivo* protein labeling experiments showed a moderately decreased synthesis rate for the chloroplast-encoded proteins, which may account for the accumulation of photosynthetic proteins (Figure 4B).

Biochemical analysis of LEPA in *E. coli* has demonstrated its function as a translation factor *in vitro*. The elongation cycle of protein synthesis is characterized by tRNA movement between pre-translocation (PRE) and post-translocation (POST) complexes. Under stress conditions, such as high salt concentration or low temperature, translocation could be blocked, possibly by perturbation of the ribosome structure [9]. LEPA could effectively compete with EFG for binding to the PRE complex. This binding could lead to the formation of an intermediate complex, I3, which could allow for the correction of an incorrect translocation event by replacing LEPA·GDP with EF-G·GTP (EF-G is present at considerably higher concentrations in bacterial cells compared with LEPA) [10]. A high Mg^2+^concentration could stabilize the I3 complex by inhibiting the conversion of I3 to a PRE complex, which explains why LEPA accelerates protein synthesis at increased Mg^2+^concentrations [6], [10]. Our study is consistent with the proposed function of LEPA as a translation factor that contributes to the efficiency of protein synthesis.

In summary, we have demonstrated the physiological role of cpLEPA in efficient photosynthesis in higher plants. In addition, we have presented evidence highlighting the importance of this protein for chloroplast translation, which provides further insights into the conserved function of LEPA in chloroplast protein synthesis.

## Materials and Methods

### Plant Material and Growth Conditions

The *cplepa-1* (T-DNA insertion line, Salk_140697) and *cplepa-2* (T-DNA insertion line, CS464145) mutants were obtained from ABRC, and the homozygous mutants were verified by PCR using the primer pairs LEPA-LP and LEPA-RP as well as LEPA-GKF+LEPA-GKR (for primer sequences, see Table S1). The T-DNA insertion was confirmed by PCR and sequencing with the primers SALKLBb1 and LEPA-LP for the *cplepa-1* mutant and with the primers GABILB and LEPA-GKR for the *cplepa-2* mutant. Wild type and mutant seeds were sterilized with 10% sodium hypochlorite for 15 min, washed five times with distilled water, and placed on solid MS medium [24] supplemented with sucrose as needed. Wild type and mutant seeds were sown and grown on soil according to a standard protocol. To ensure synchronized germination, the seeds were kept in the dark at 4°C for two days. The *Arabidopsis* plants were kept in a growth chamber at 22°C with a 12-h photoperiod at a photon flux density of 120 µmol m^−2^ s^−1^.

### Photoinhibitory Treatment

Detached wild type and *cplepa-1* mutant leaves were floated face down on water and illuminated under a photon flux density of 1,000 µmol m^−2^ s^−1^, and the chlorophyll fluorescence was measured at 0.5 h, 1 h, 2 h, 3 h, and 4 h after exposure to high light using a PAM-2000 fluorometer (Walz). The temperature was maintained at 22°C throughout the photoinhibitory treatments. The synthesis of chloroplast-encoded proteins was blocked by incubating detached leaves with 1 mM lincomycin at low light (20 µmol m^−2 ^s^−1^) for 3 h before photoinhibition treatment. To investigate the effects of high light on plant growth, we transferred 2-week-old *Arabidopsis* plants grown on soil under normal illumination of 120 µmol m^−2^ s^−1^ to 500 µmol m^−2^ s^−1^for another 2 weeks.

### Complementation

To complement the *cpLEPA* mutation, a full-length *cpLEPA* cDNA was amplified using nested antisense primers (LEPAH-F, LEPAH-R1 and LEPAH-R2) with HIS tags, and the product was subcloned into the pSN1301 vector under the control of the CAMV 35S promoter. The constructed plasmids were then transformed into *Agrobacterium tumefaciens* strain *C58* and introduced into the *cplepa-1* mutant plants by a floral dip method, as described previously [25]. Transgenic plants were selected on MS medium containing 50 µg/mL hygromycin. Complemented plants were selected and transferred to soil to produce seeds. The success of the complementation was confirmed by PCR, immunoblot and chlorophyll fluorescence analysis.

### Chloroplast Ultrastructure

Wild type and mutant leaves from 3-week-old plants grown on soil were used for transmission electron microscopy analysis. The leaves were chopped into 1×2 mm pieces and immersed in fixative solution (2.4% glutaraldehyde in phosphate buffer) for 4 h at 4°C. After fixation, the samples were rinsed and postfixed in 1% OsO_4_ overnight at 4°C and then dehydrated in an ethanol series, infiltrated with a graded series of epoxy resin in epoxy propane, and embedded in Epon 812 resin. Thin (80–100 nm) sections were obtained using a diamond knife on a Reichert OM2 ultramicrotome. The sections were stained with 2% uranyl acetate, pH 5.0, followed by 10 mM lead citrate, pH 12, and observed with a transmission electron microscope (Jem-1230; JEOL).

### *In vivo* Protein Labeling Assays

*In vivo* protein labeling was performed essentially according to Meurer *et al* [26]. For pulse labeling, primary leaves from 12-d-old plants were labeled with 1 µCi/µL [^35^S]-Met in the presence of 20 µg/mL cycloheximide for 20 min at 25°C. After labeling, the leaves were washed twice with homogenization buffer (50 mM Tris-HCl, pH 7.5, 150 mM NaCl, and 2 mM EDTA) and ground with 300 µL of the same buffer. The thylakoid membranes were isolated and separated by SDS-PAGE, and the labeled proteins were visualized by autoradiography.

### Chloroplast and Thylakoid Membrane Preparation

Intact chloroplasts were fractionated into envelope, stromal and thylakoid membrane fractions as described previously [27]–[29]. The thylakoid membranes were isolated according to Cai *et al* [30]. The chlorophyll contents were measured as described previously [31].

### Protein Localization Analysis

The thylakoid membranes from wild type plants were suspended to a final concentration of 0.1 mg chlorophyll/mL in 10 mM HEPES-KOH, Ph 8.0, 10 mM MgCl_2_, 330 mM sorbitol, and 1 mM PMSF supplemented with either 250 mM NaCl, 1 M CaCl_2_, 200 mM Na_2_CO_3_ or 6 M urea. The membrane fractions without treatment were used as controls. All of the samples were kept on ice during the experiment. The treated samples were washed with 10 mM HEPES-KOH, pH 8.0, 10 mM MgCl_2_, 330 mM sorbitol, and 1 mM PMSF, and the pellets were collected by centrifugation for western blot analysis [32], [33].

### Immunoblot Analysis

Total protein was extracted from 3-week-old wild-type and mutant plants using E buffer (125 mM Tris-HCl, pH 8.8; 1% (w/v) SDS; 10% (v/v) glycerol; 50 mM Na_2_S_2_O_5_) as described by Martínez-García *et al* [34]. Protein concentration was determined using the BioRad Dc Protein Assay (BioRad, Hercules, CA, USA) according to the manufacturer’s instructions. Total proteins were separated by SDS-PAGE and transferred onto nitrocellulose membranes. After incubation with specific primary antibodies, the signals from secondary conjugated antibodies were detected by the enhanced chemiluminescence method. The anti-cpLEPA antibody was raised against the N-terminus of the cpLEPA protein (cpLEPA_56–170_). The procedures involved in generating an antibody were performed according to Sun *et al* [35].

### RT-PCR, Northern Blot and Polysome Association Analyses

For the RT-PCR analysis, the total RNA was isolated from 3-week-old leaves using the Total RNA Isolation Kit (U-Gene), and RT-PCR was performed with the SuperScript III First-Strand Synthesis System for RT-PCR (Invitrogen) using the primers LEPA RTF and LEPA RTR.

For northern blot analysis, total RNA was extracted from 3-week-old wild type and mutant plants after germination on MS or soil as described above. The northern blot was performed according to Cai *et al* [36]. The following primer pairs were used to amplify the appropriate probes: *psbA*, *psbB*, *psbD*, *atpB*, *petB*, *rbcL*, *psaA*, *rrn23*, *rpoA*, *rpoB, ndhA*, *petA* and *psaJ* (Table S1 for primer sequence).

For polysome association analysis, polysomes were isolated from 3-week-old leaves according to Barkan [37], with certain modifications. Less than 0.3 g of leaf tissue was frozen and ground in liquid nitrogen to a fine powder, 1 mL of polysome extraction buffer (0.2 M Tris-HCl, pH 9; 0.2 M KCl, 35 mM MgCl_2_, 25 mM EGTA, 0.2 M sucrose, 1% Triton X-100, 2% polyoxyethylene-10-tridecyl ether, 0.5 mg/mL heparin, 100 mM β-mercaptoethanol, 100 µg/mL chloramphenicol, and 25 µg/mL cycloheximide) was added, and the tissue was ground until thawed. The samples were incubated on ice for 10 min and pelleted by centrifugation for 7 min at 14,000 rpm. Sodium deoxycholate was added to the supernatant to a final concentration of 0.5%, after which the samples were kept on ice for 5 min and then centrifuged at 12,000 rpm for 15 min. Next, 0.5 mL samples of the supernatant were layered onto 4.4-mL sucrose gradients that were prepared, centrifuged, and fractionated as described previously [37]. The samples were kept at 4°C during preparation. A crude polysome sample supplemented with 20 mM EDTA was analyzed in parallel on a similar gradient containing 1 mM EDTA instead of MgCl_2_ . The RNA in each fraction was isolated, separated and transferred onto nylon membranes (Amersham Phamacia Biotech), which were probed with ^32^P-labeled probes prepared according to Cai *et al* [36] and exposed to x-ray films.

## Supporting Information

Figure S1 **Transmission Electron Micrographs of the Chloroplasts.** Transmission electron microscopic images of the chloroplast ultrastructure in WT and *cplepa-1* leaf sections. Three-week-old plants grown on soil at 120 µmol m^−2^ s^−1^ were used. The scale bar indicates 1 µm. In total, 100 chloroplasts of the WT and *cplepa-1* were examined and measured.
(TIF)

Figure S2 **Polysome Association Analysis for Chloroplast Transcripts in Wild-Type and** ***cplepa-1*** **Plants Grown in Soil.** A: The association of *rrn23* and *rbcL* transcripts with EDTA treated polysomes. Crude leaf lysates treated with 20 mM EDTA from wild-type were size fractionated on 15% to 55% sucrose gradients containing 1 mM EDTA. B: The association of *ndhA*, *petA* and *psaJ* transcripts with polysomes. Total extracts from wild-type and *cplepa-1* leaves grown on soil for 3 weeks at 120 µmol m^−2^ s^−1^ were fractionated on 15%–55% sucrose gradients. The rRNAs were detected by ethidium bromide (EtBr) staining. The size of the transcript (in kb) is shown.
(TIF)

Figure S3 **Polysome Association Analysis of Chloroplast Transcripts in Wild-Type and** ***cplepa-1*** **Plants Grown on MS.** The association of the *psbA*, *psbB*, *atpB*, *psaA*, *petB* and *rrn23* transcripts with polysomes. Total extracts from wild-type and *cplepa-1* leaves grown on MS solid medium supplied with 2% sucrose for 3 weeks under 120 µmol m^−2^ s^−1^ illumination were fractionated on 15%–55% sucrose gradients. Ten fractions of equal volume were collected from the top to the bottom of the sucrose gradients, and equal proportions of the RNA purified from each fraction were analyzed by northern blot. The rRNAs were detected by ethidium bromide (EtBr) staining. The size of the transcript (in kb) is shown.
(TIF)

Figure S4 **Northern Blot Analysis for Chloroplast Transcripts in Wild-Type and** ***cplepa-1*** **Plants.** Northern blot analysis of chloroplast transcripts *psbA*, *psbB*, *psbD*, *atpB*, *psaA*, *petB*, *rbcL*, and *rrn23* in wild-type and *cplepa-1* mutant plants. The lanes were loaded with 10 µg total RNA each. Wild-type and *cplepa-1* were grown on MS solid medium supplied with 2% sucrose for 3 weeks under 120 µmol m^−2^ s^−1^ illumination. Additionally, 25S rRNA stained with EtBr was loaded as a control. The size of the transcript (in kb) is shown.
(TIF)

Table S1 Primer sequences and probes used in this work.
(DOC)
