# Supplementary figures and images for: CpLEPA Is Critical for Chloroplast Protein Synthesis Under Suboptimal Conditions in Arabidopsis thaliana
Source: PLoS One. 2012 Nov 15;7(11):e49746. doi: 10.1371/journal.pone.0049746 (PMC3499520; doi:10.1371/journal.pone.0049746)

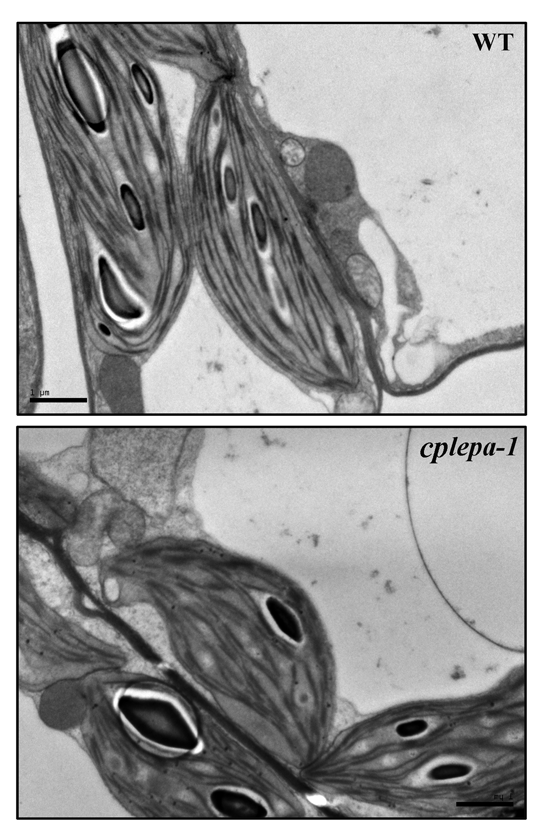

Supplement: Figure S1 — Transmission Electron Micrographs of the Chloroplasts. Transmission electron microscopic images of the chloroplast ultrastructure in WT and cplepa-1 leaf sections. Three-week-old plants grown on soil at 120 µmol m−2 s−1 were used. The scale bar indicates 1 µm. In total, 100 chloroplasts of the WT and cplepa-1 were examined and measured. (TIF) [file pone.0049746.s001.tif]

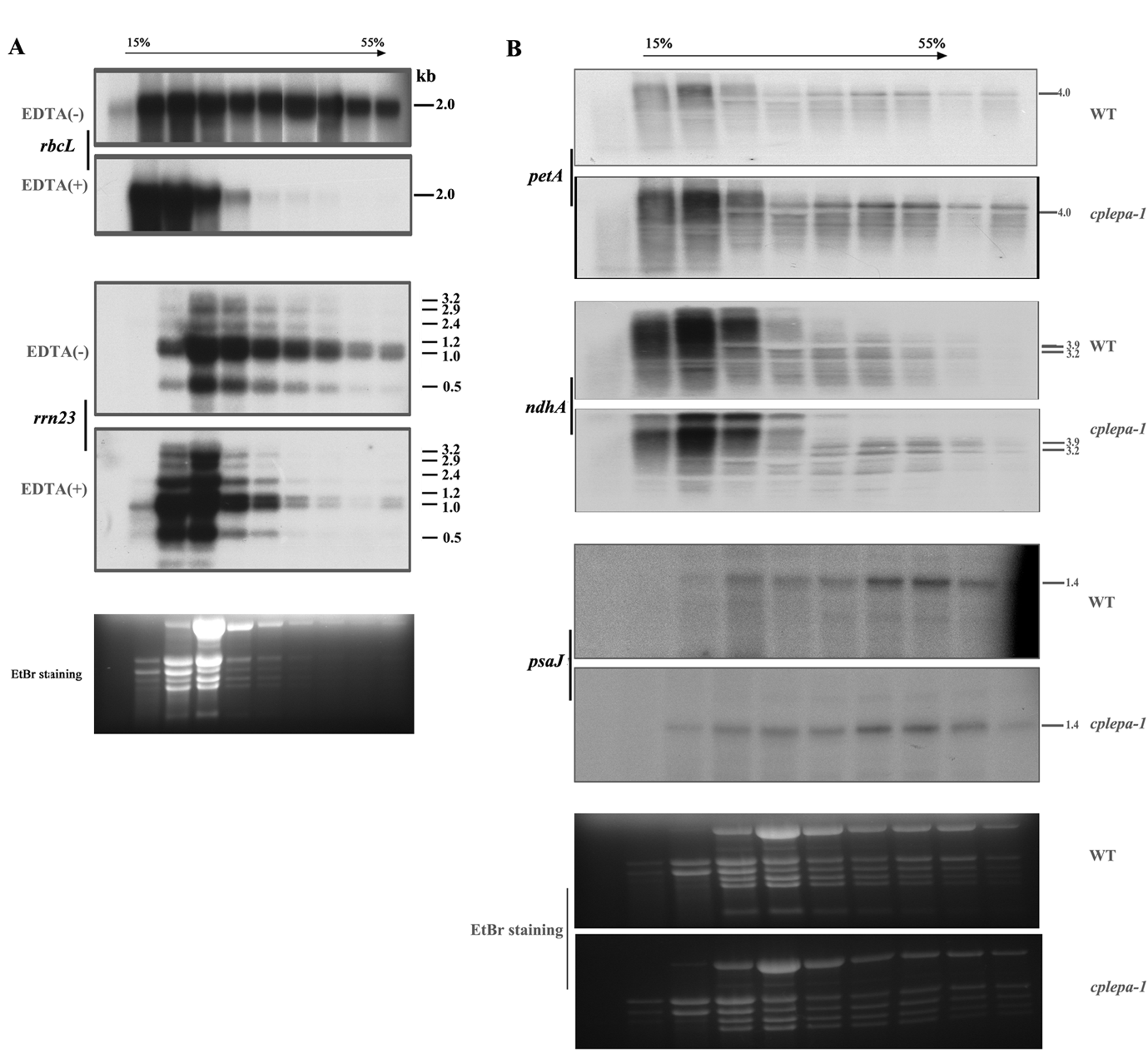

Supplement: Figure S2 — Polysome Association Analysis for Chloroplast Transcripts in Wild-Type and cplepa-1 Plants Grown in Soil. A: The association of rrn23 and rbcL transcripts with EDTA treated polysomes. Crude leaf lysates treated with 20 mM EDTA from wild-type were size fractionated on 15% to 55% sucrose gradients containing 1 mM EDTA. B: The association of ndhA, petA and psaJ transcripts with polysomes. Total extracts from wild-type and cplepa-1 leaves grown on soil for 3 weeks at 120 µmol m−2 s−1 were fractionated on 15%–55% sucrose gradients. The rRNAs were detected by ethidium bromide (EtBr) staining. The size of the transcript (in kb) is shown. (TIF) [file pone.0049746.s002.tif]

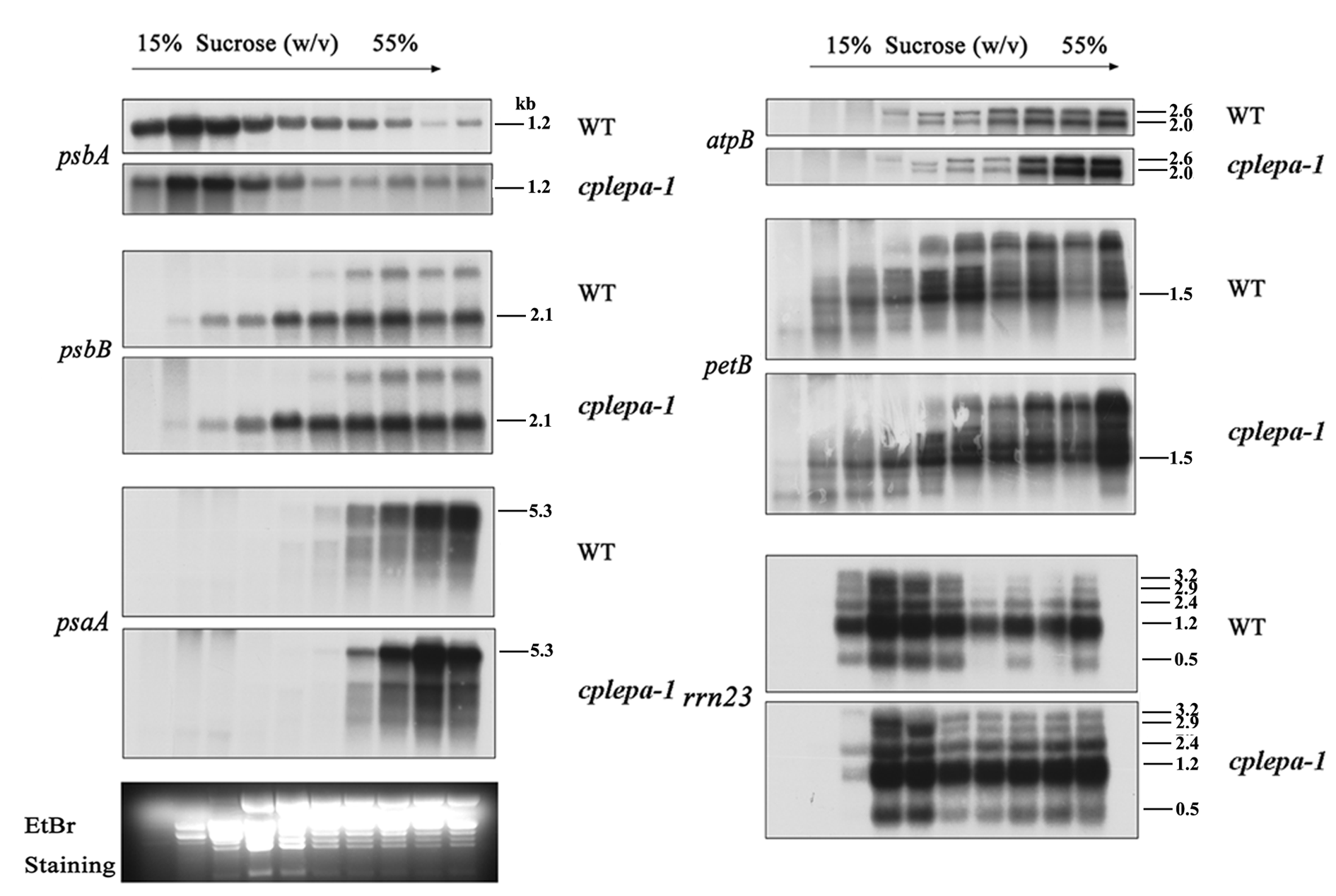

Supplement: Figure S3 — Polysome Association Analysis of Chloroplast Transcripts in Wild-Type and cplepa-1 Plants Grown on MS. The association of the psbA, psbB, atpB, psaA, petB and rrn23 transcripts with polysomes. Total extracts from wild-type and cplepa-1 leaves grown on MS solid medium supplied with 2% sucrose for 3 weeks under 120 µmol m−2 s−1 illumination were fractionated on 15%–55% sucrose gradients. Ten fractions of equal volume were collected from the top to the bottom of the sucrose gradients, and equal proportions of the RNA purified from each fraction were analyzed by northern blot. The rRNAs were detected by ethidium bromide (EtBr) staining. The size of the transcript (in kb) is shown. (TIF) [file pone.0049746.s003.tif]

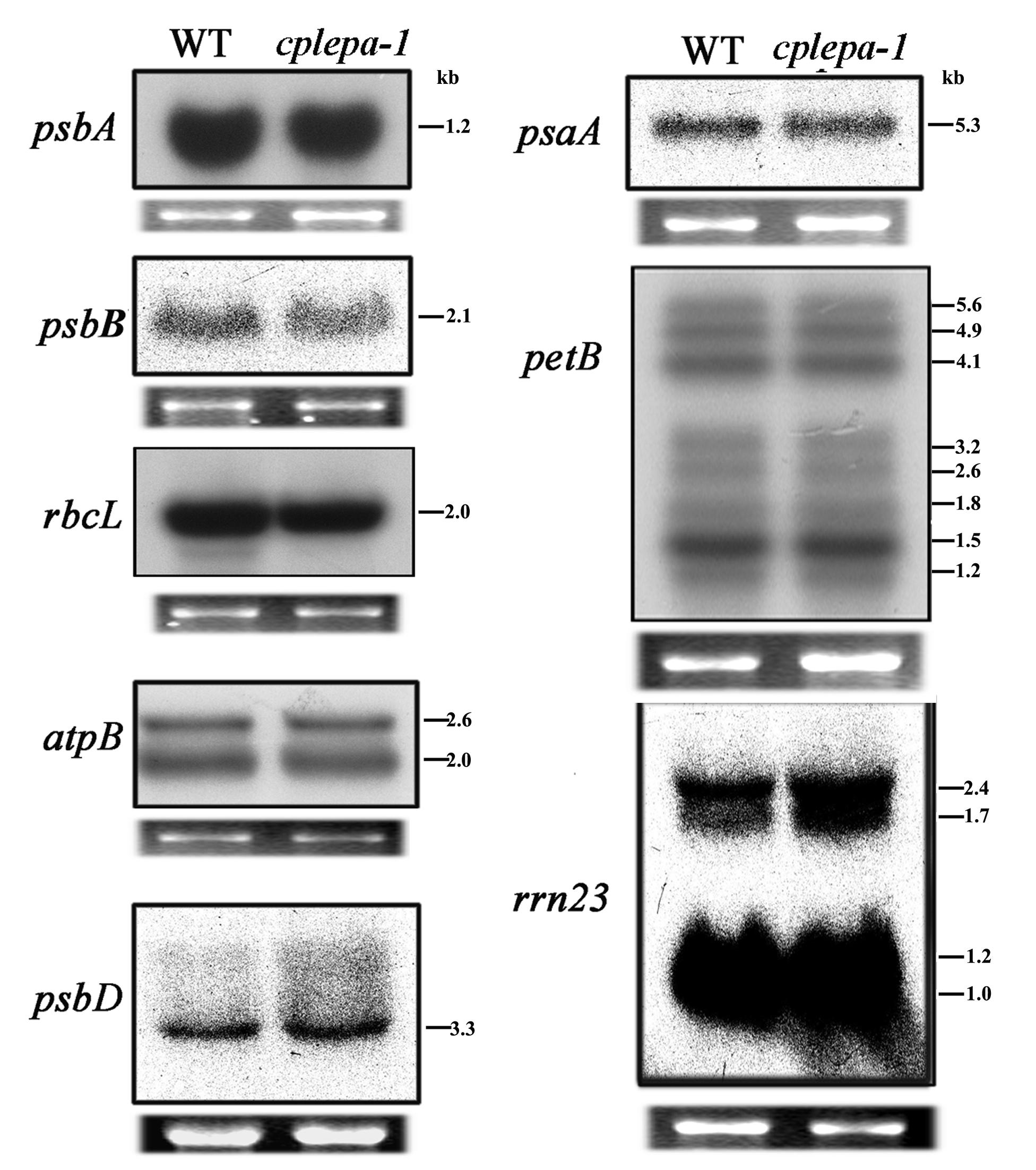

Supplement: Figure S4 — Northern Blot Analysis for Chloroplast Transcripts in Wild-Type and cplepa-1 Plants. Northern blot analysis of chloroplast transcripts psbA, psbB, psbD, atpB, psaA, petB, rbcL, and rrn23 in wild-type and cplepa-1 mutant plants. The lanes were loaded with 10 µg total RNA each. Wild-type and cplepa-1 were grown on MS solid medium supplied with 2% sucrose for 3 weeks under 120 µmol m−2 s−1 illumination. Additionally, 25S rRNA stained with EtBr was loaded as a control. The size of the transcript (in kb) is shown. (TIF) [file pone.0049746.s004.tif]
