# Supplementary material for: CpLEPA Is Critical for Chloroplast Protein Synthesis Under Suboptimal Conditions in Arabidopsis thaliana
Source: PLoS One. 2012 Nov 15;7(11):e49746. doi: 10.1371/journal.pone.0049746 (PMC3499520; doi:10.1371/journal.pone.0049746)
Supplement: Table S1 — Primer sequences and probes used in this work. (DOC) [file pone.0049746.s005.doc]

**Table S1. Primer Sequences and Probes Used in This Work.**

| **Name** | **Sequence 5’-3’** | | | **Experiment** |
| --- | --- | --- | --- | --- |
| LEPA-LP | TGAAGATACACCGTTTTGCCTC | | | T-DNA insertion |
| LEPA-RP | ACAATTACCCCATTCGAGGAAC | | |  |
| SALKLBb1 | GCGTGGACCGCTTGCTGCAACT | | |  |
| LEPA RTF | GAACTTCTACATCTTCCCCATC | | | RT PCR |
| LEPA RTR | TCACCCTATAAACAACACTTGG | | |  |
| LEPA -GKF | CAGGATTATTTTGCGGATGAAGTC | | | T-DNA insertion |
| LEPA- GKR | ACAATTTCCATGTGGAGAAGACCC | | |  |
| GABI-LB | ATATTGACCATCATACTCATTGC | | |  |
| LEPAH-F | CCCGGGATGGCCATGGCTTCTGCTAT GGACTTATC | | | complementation |
| LEPAH-R1 | GTGGTGGTGGTGCAATACTTCTCGTTCAAGTTTGAG | | |  |
| LEPAH-R2 | GGTACCCTAGTGGTGGTGGTGGTGGTGCAATACTTC | | |  |
| atp*B-F* | GGGGAACCCGTTGATAATTT | | | probe *atpB* |
| atp*B-R* | AACGCTCAATTTTTCGTGCT | | |  |
| *petB-F* | ATTGTCTAGGCGGAATTACCC | | | probe *petB* |
| *petB--R* | CCAGAAATACCTTGCTTACGTATC | | |  |
| *psaA-F* | CAATTGGCGCATTGGTCTTCGCAG | | | probe *psaA* |
| *psaA-R* | GTGCTCGCTGTTTCACCAGGGGCTG | | |  |
| *psbA-F* | ATGACTGCAATTTTAGAGAGACGCG | | | Probe  *psbA* |
| *psbA-R* | TTATCCATTTGTAGATGGAGCCTCA | | |  |
| *psbB-F* | GCAAGGATCCATGGGTTTGCCTTGG | | | probe *psbB* |
| *psbB-R* | GCAACTCGAGATCAGACTGCTTGTCG | | |  |
| *psbD-F* | CTGGTCTATTGCTCTTTCCTTGTGCCTAT | | | probe *psbD* |
| *psbD-R* | GCCGCCATCCAAGCACGAATACCTT | | |  |
| *rbcLU-F* | AAGTGTTGGGTTCAAAGCTG | | | probe *rbcL* |
| *rbcLD-R* | GGCCATCTAATTTATCGATGGT | | |  |
| *rrn23-F* | TTCAAACGAGGAAAGGCTTA | | | probe *rrn23* |
| *rrn23-R* | AGGAGAGCACTCATCTTGGG | | |  |
| *RpoaF* | ATGGTTCGAGAGAAAGTCAAAG | | | Probe *rpoA* |
| *RpoaR* | TTGACTGTTATTCAAAAGGTCC | | |  |
| *RpobF* | ATGGTGCTGCTACAGTTGGT | | | Probe *rpoB* |
| *RpobR* | CAAACCTCCATTTCGCCTAC | | |  |
| PSAJF | ATGCGAGATC TAAAAACATA | | | Probe  *psaJ* |
| PSAJR | CTAGAATGAAAAAAAGGGAAATG | | |  |
| RbcLF | AAGTGTTGGGTTCAAAGCTG | | | Probe *rbcL* |
| RbcLR | GGCCATCTAATTTATCGATGGT | | |  |
| NDHAF | ATGATAATTT ATGCAACAGC | | | Probe *ndhA* |
| NDHAR | TTAGAGTGAAAAGAGTTGGAAAG | | |  |
| PETAF | ATGCAAACTA GAAATACCTT TTC | | | Probe *petA* |
| PETAR | CTAAAAATTCATTTCGGATAATT | | |  |
|  | |  |  | |
